# A happy medium: the synthesis of medicinally important medium-sized rings *via* ring expansion

**DOI:** 10.1039/d0sc00568a

**Published:** 2020-03-02

**Authors:** Aimee K. Clarke, William P. Unsworth

**Affiliations:** Department of Chemistry, University of York York YO10 5DD UK william.unsworth@york.ac.uk

## Abstract

Medium-sized rings have much promise in medicinal chemistry, but are difficult to make using direct cyclisation methods. In this minireview, we highlight the value of ring expansion strategies to address this long-standing synthetic challenge. We have drawn on recent progress (post 2013) to highlight the key reaction design features that enable successful ‘normal-to-medium’ ring expansion for the synthesis of these medicinally important molecular frameworks, that are currently under-represented in compound screening collections and marketed drugs in view of their challenging syntheses.

## Abstract 2

Ring expansion strategies are ideally suited to make synthetically challenging, medium-sized rings with much potential in medicinal chemistry.

## Graphical abstract

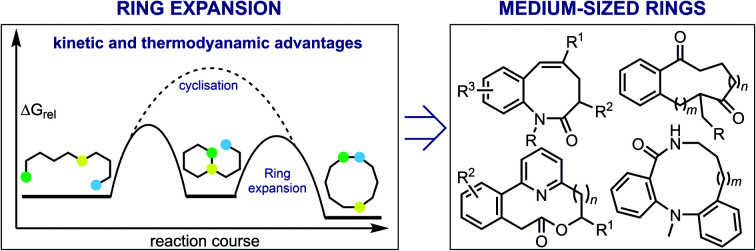

## Introduction

**Fig. 1:**
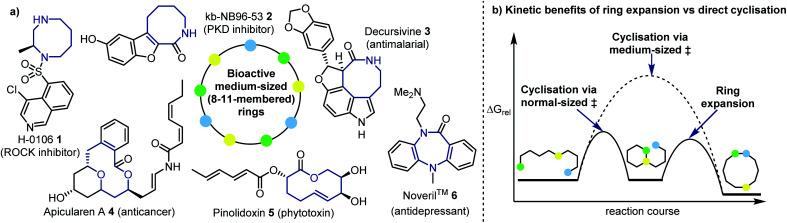
(a) Bioactive medium-sized rings. (b) Kinetic benefits of ring expansion compared with direct cyclisation.

Medium-sized rings are commonly found in diverse bioactive natural products and therapeutically important molecules (*e.g.***1–6**, Fig. 1a).^1,2^ Distinct from normal-sized rings (5–7-membered) and macrocycles (12+ membered rings),^3^ the relative rigidity and diverse 3D spatial properties of medium-sized (8–11-membered) rings often results in improved binding affinity to biological receptors, oral bioavailability and cell permeability, compared with analogous acyclic molecules or other rings sizes.^4^ These attractive features have helped drive a real surge in interest in both the synthesis and applications of medium-sized rings and macrocycles for medicinal purposes,^1–4^ both within academia and the pharmaceutical industry.

However, despite their promise, medium-sized rings are underrepresented in marketed drugs and drug discovery programmes, largely due to the well-known challenge of making them.^5^ Medium-sized rings occupy a somewhat unfortunate space synthetically, where the kinetic and thermodynamic barriers associated with their synthesis are typically higher than for other rings sizes; *i.e.* they are sufficiently large that synthesis *via* cyclisation of a linear precursor is often associated with significant loss of entropy, but small enough to experience destabilising transannular interactions and strain.^6^

Understanding these thermodynamic challenges is crucial when designing new strategies to make medium-sized rings. One strategy is to ‘grow’ medium-sized rings *via* ring expansion reactions,^7^ which means that the kinetic challenges associated with direct cyclisation approaches (*e.g.* metathesis, acylation, cycloaddition *etc.*)^8^ can be avoided (see Fig. 1b for a stylised reaction coordinate comparing the two scenarios). However, for such approaches to be successful, it is vital that the reaction is designed in such a way as to provide a clear thermodynamic imperative for rearrangement to occur; based on ring size alone, the conversion of normal- into medium-sized rings is usually always ‘uphill’ thermodynamically,^6^ thus, for ring expansion reactions to be exergonic overall, this thermodynamic cost must be ‘repaid’ *via* some other means.

The most common ways that this can be achieved are summarised in this minireview, in which we have particularly focused on exemplifying the key design features that drive the successful outcomes. Of course, it has been known for decades that ring expansion can be a powerful tool for the synthesis of medium-sized rings (and macrocycles), with the eponymous reactions developed by Grob and Eschenmoser,^7*e*–*g*^ and the many important works of Manfred Hesse,^7*b*,7*h*–*k*^ being especially noteworthy. These seminal studies and others have featured in various previous review articles,^7*a*–*c*^ therefore, in this minireview, it was decided to focus on more recent (post 2013) state-of-the-art ring expansion methods for the synthesis of medium-sized rings. The material in this minireview is separated into 5 main sections (A–E) based on the thermodynamic features that help drive ring expansion. It is not intended to provide a comprehensive coverage, but rather, provide a balanced summary of recent methods that best exemplify each of the key thermodynamic driving forces. To provide a simple, easy to visualise representation of the reaction scope with respect to ring size, the ring sizes accessed to date using each method are highlighted (light blue).

### (A) Bond dissociation energies

**Scheme 1:**
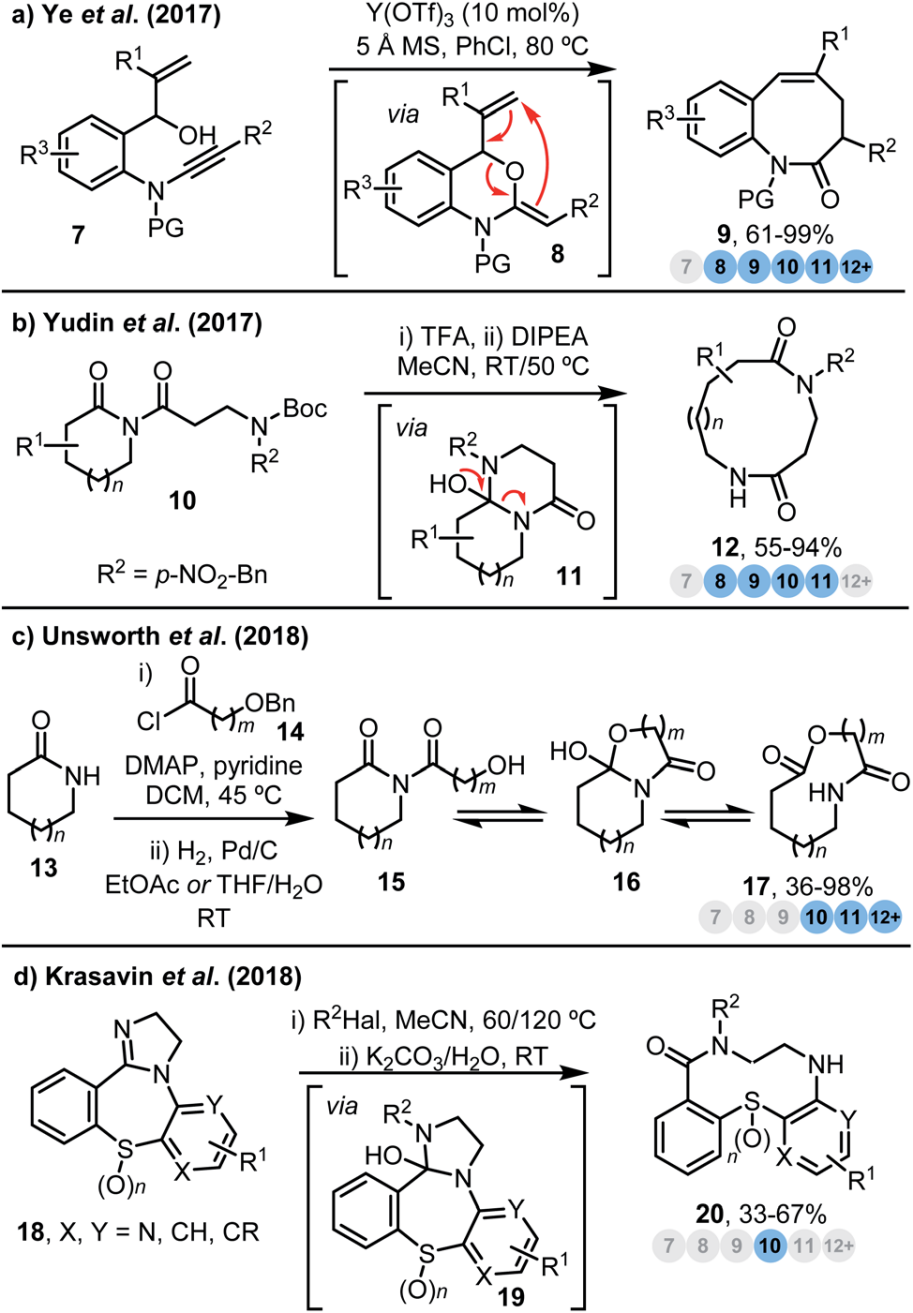
Ring expansion strategies driven by the formation of thermodynamically favourable bonds. (a) Ye *et al.*^9*a*^ (b) Yudin *et al.*^10^ (c) Unsworth *et al.*^11^ (d) Krasavin *et al.*^13*b*^

Many successful ring expansion reactions are driven by favourable changes in the overall bonding in the ring expanded products compared to the respective starting material(s); *i.e.* by using chemical energy contained within relatively reactive functional groups to promote ring expansion *via* conversion into more thermodynamically stable species (*e.g.* amides – as is demonstrated in all four reactions highlighted in this section). An instructive example of this approach is found in a 2017 report by Ye and co-workers,^9*a*^ who reported an efficient yttrium-catalysed intramolecular hydroalkoxylation/Claisen rearrangement sequence to access a range of medium-sized lactams in good to excellent yields (**7** → **9**, Scheme 1a). The authors propose that π-acid activation of ynamide alkyne **7** promotes the formation of a reactive intermediate **8**, which then undergoes a [3,3]-sigmatropic rearrangement^9^ to furnish ring expanded products **9**. The formation of a strong C=O (amide) bond in lactams **9** presumably helps the ring expansion to proceed.

Amide formation is also an important driving force for ring expansion in a protocol reported by Yudin and co-workers,^10^ whereby a range of β-amino imides **10** can be converted into their corresponding bis lactams **12***via* the expansion of cyclol intermediates **11** (Scheme 1b). In the same study, the conversion of 2,5-diketopiperazines into cyclic tripeptides is also reported (not shown). Computational (DFT) analysis of the isomers involved in the key rearrangement step was performed, and the importance of relative energies of each species in the ring expansion equilibrium is demonstrated by the observation that conversion into the ring expanded product (*e.g.***12**) only occurs successfully if this product is ‘exergonic by at least 6–12 kcal mol^−1^’, when compared to the cyclol intermediate (*e.g.***11**).

The importance of the relative energies of isomeric species was also noted in a conceptually related ring expansion process from our own laboratory, in which imides can be converted into ring expanded lactones (**15** → **16** → **17**, Scheme 1c),^11^ with this work being part of a wider programme of research on lactam/lactone synthesis using Successive Ring Expansion (SuRE) reactions.^12^ As was the case in Yudin's study,^10^ ring size was pivotal in determining the reaction outcome, with ring expansion only viable for the synthesis of lactones above a certain ring size. The reactions were also studied using DFT, the results of which fully support the notion that these ring expansion reactions are under thermodynamic control; indeed, this work is a useful illustration of the important role ring size plays in influencing the thermodynamic outcomes of ring expansion reactions more broadly.

Krasavin *et al.* reported the hydrolytic imidazoline ring expansion (HIRE) of thiazepines and their derivatives, that enables the facile formation of medium-sized thiadiazecines (Scheme 1d).^13*a*^ The reaction proceeds *via* initial activation of the 2-imidazoline moiety by *N*-alkylation, to generate an imidazolinium salt, which promotes nucleophilic attack by water to form a cyclol intermediate **19** under basic conditions. This species then undergoes ring expansion to form products **20**, in analogy to Yudin's and our own methods.^10–12^ The same group also extended this work to prepare a range of similar medium-sized rings *via* a side-chain insertion ring expansion methodology proceeding *via* the same type of hydrated cyclol intermediates (*cf.***19**) as seen in their previous work.^13*b*,14^

### (B) Charged intermediates

**Scheme 2:**
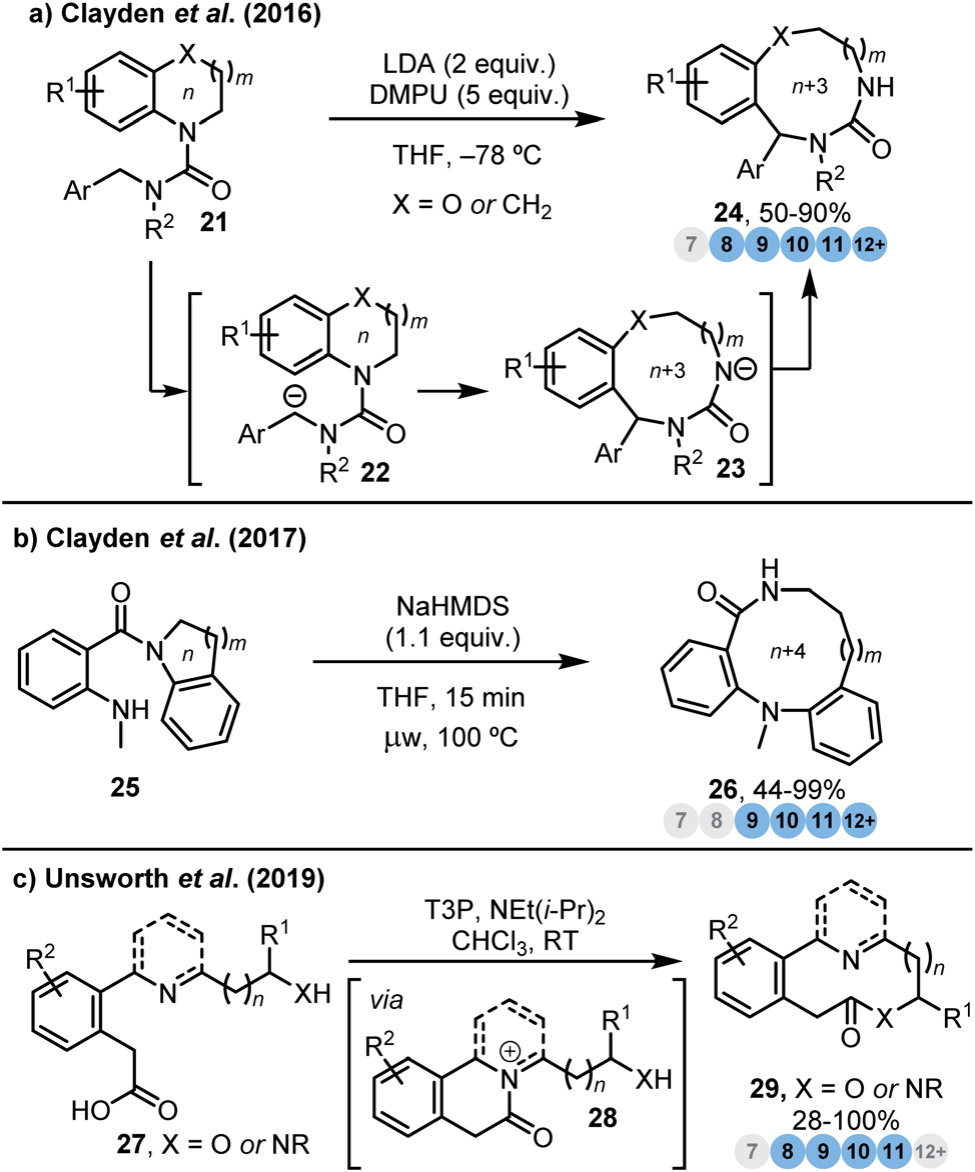
Ring expansion strategies driven by the neutralisation or stabilisation of charged reactive intermediates. (a) Clayden *et al.*^15*a*^ (b) Clayden *et al.*^15*b*^ (c) Unsworth *et al.*^16*a*^

The neutralisation or stabilisation of charged reactive intermediates can also serve as a driving force for ring expansion. In 2016, Clayden and co-workers reported a migratory ring expansion methodology through deprotonation of benzylic urea derivatives **21** to rapidly generate complex 8–12-membered benzodiazepines, benzodiazocines and their homologues **24** (Scheme 2a).^15*a*^ Here, the *n* → *n* + 3 ring expansion benefits from the stabilisation of a negative charge, as reactive carbanion **22** rearranges into the comparatively stabilised urea anion **23**. The same group also reported the use of an unconventional variant of the Smiles rearrangement to generate medium-sized rings **26** from benzo-fused nitrogen heterocycles **25** (Scheme 2b) *via* an *n* → *n* + 4 ring expansion,^15*b*^ which also benefits from anion stabilisation during the ring expansion step.

More recently, our group developed a cyclisation/ring expansion cascade process^16*a*^ facilitated by an internal nucleophilic catalyst, to furnish medium-sized lactams and lactones directly from linear precursors (Scheme 2c).^16^ A key feature of these cascades is that they proceed exclusively *via* ‘normal’-sized cyclic transition states, and avoid medium-sized transition states. It is proposed that following activation of the carboxylic acid, a tertiary amine strategically placed within the linear starting material **27**, acts as an internal nucleophilic catalyst, resulting in the rapid formation of an acyl ammonium ion intermediate **28**. This intermediate is not observed, but rather undergoes spontaneous ring expansion, to deliver the final ring expanded lactone/lactam products **29**. Neutralisation of the positive charge in **28** presumably plays a key role in providing a thermodynamic driving force for the normal-to-medium-sized rearrangement (8–11-membered rings can all be made using this method).

### (C) Aromatisation

**Scheme 3:**
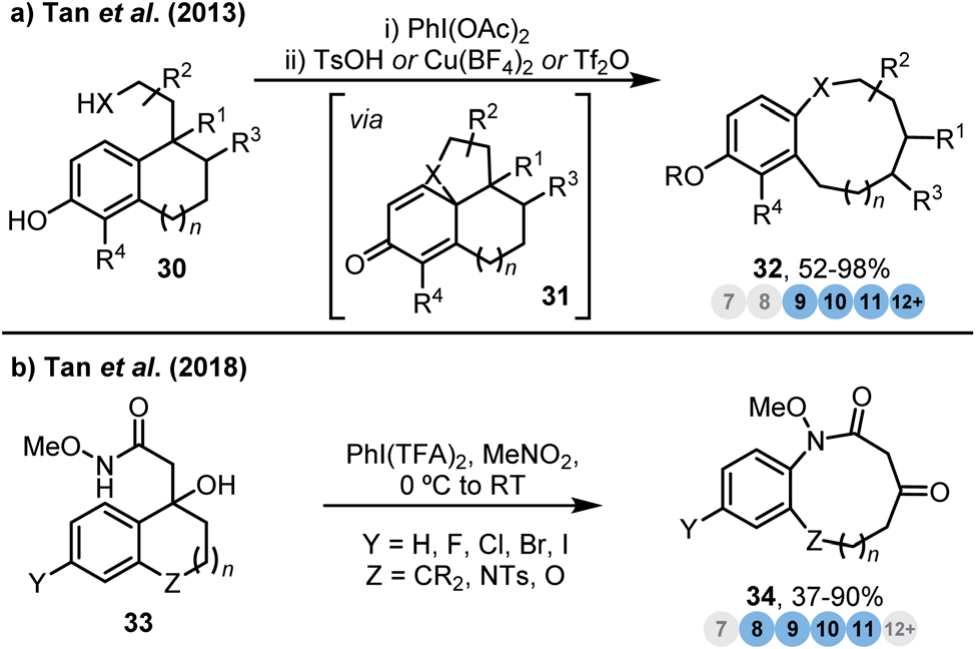
Ring expansion strategies promoted by the formation of stable aromatic products. (a) Tan *et al.*^17*a*^ (b) Tan *et al.*^17*b*^

Ring expansion reactions can also be promoted by the formation (or re-formation) of stable aromatic products. Tan and co-workers developed a biomimetic diversity-oriented synthesis of benzannulated medium-sized rings *via* oxidative dearomatisation ring expansion (ODRE) of phenols (Scheme 3a).^17*a*^ Initial oxidative dearomatisation of phenols **30** affords polycyclic cyclohexadienone intermediates **31** that undergo aromatisation-driven ring expansion, to deliver a variety of medium-sized ring scaffolds **32** found in natural products. The reaction can be induced by three complementary reagents (TsOH, Cu(BF_4_)_2_ or Tf_2_O) that avoid competing dienone–phenol rearrangements, although sometimes olefin isomers and solvent adducts can arise from other termination pathways. To overcome these limitations, the same group later reported an improved tandem ODRE reaction that utilises a novel Umpolung approach, whereby the initial oxidative dearomatisation proceeds *via* attack of an electron-rich aromatic onto an electrophilic side chain (Scheme 3b).^17*b*^ This adaptation enabled the use of a wider range of non-phenolic substrates and heteroaromatics (not shown), and also enables some of the previously reported unwanted termination pathways to be avoided, thus leading to the formation of ketone products **34**. Rearomatisation of the initially oxidised moiety in the ring expansion step is likely to play a key part in both transformations, with each having demonstrable potential for the exploration of 3D drug-like space.

### (D) Ring strain

**Scheme 4:**
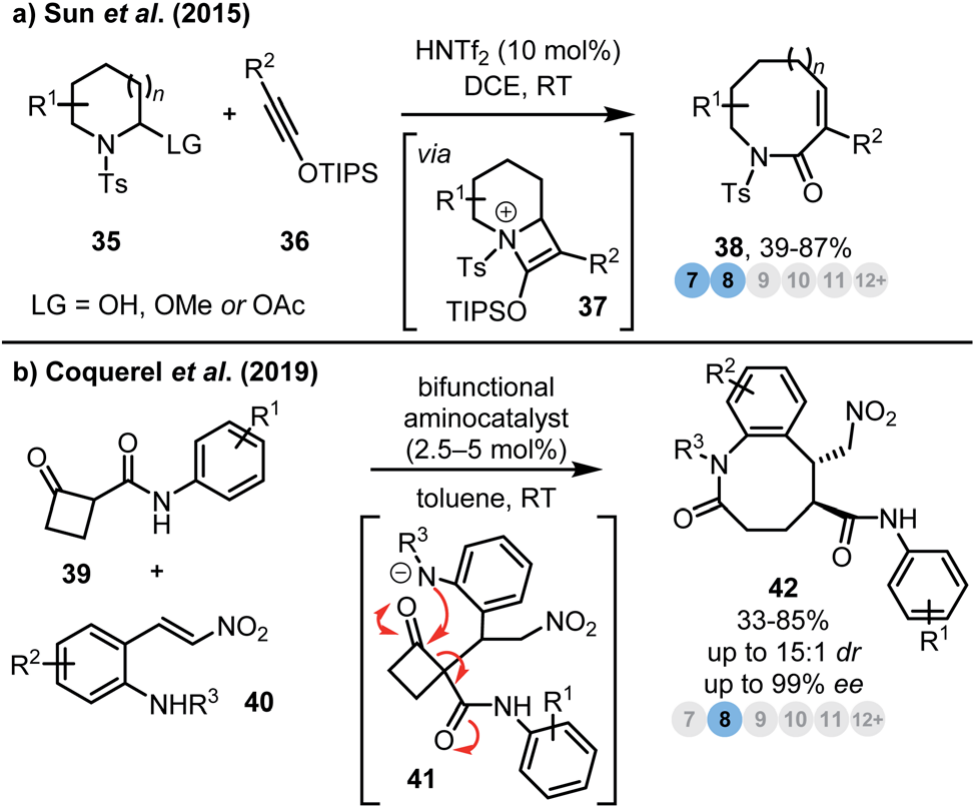
Ring expansion strategies driven by the relief of ring strain. (a) Sun *et al.*^18^ (b) Coquerel *et al.*^19^

Relief of ring strain (especially in 4-membered rings) can also be used to drive ring expansion reactions. In 2015, Sun and co-workers reported an efficient catalytic ring expansion procedure using *N*-sulfonyliminiums **35** and siloxy alkynes **36** to prepare a range of medium-sized lactams **38** (Scheme 4a).^18^ The authors propose that the reaction proceeds *via* initial acid-catalysed activation of *N*-sulfonyliminium **35***via* loss of a water/alcohol leaving group, to generate an iminium species that goes on to react with siloxy alkyne **36** to furnish azetidinium **37**. This strained (and charged) reactive intermediate is then primed to undergo ring expansion, thereby releasing ring strain and delivering the desired ring expanded products **38**.

Most organocatalytic cascade reactions focus on the preparation of more energetically favourable 5- and 6-membered rings; however Coquerel *et al.* devised an innovative enantioselective organocatalytic synthesis of benzazocinones **42** based on a Michael addition-4-atom ring expansion cascade from activated cyclobutanones **39** and *ortho*-amino nitrostyrene derivatives **40** (Scheme 4b).^19^ Relief of ring strain means that even relatively stabilised anilide nucleophiles are sufficiently reactive to promote effective 4- to 8-membered ring expansion, *via* cyclobutanone intermediates of the form **41**.

### (E) Radical stability

**Scheme 5:**
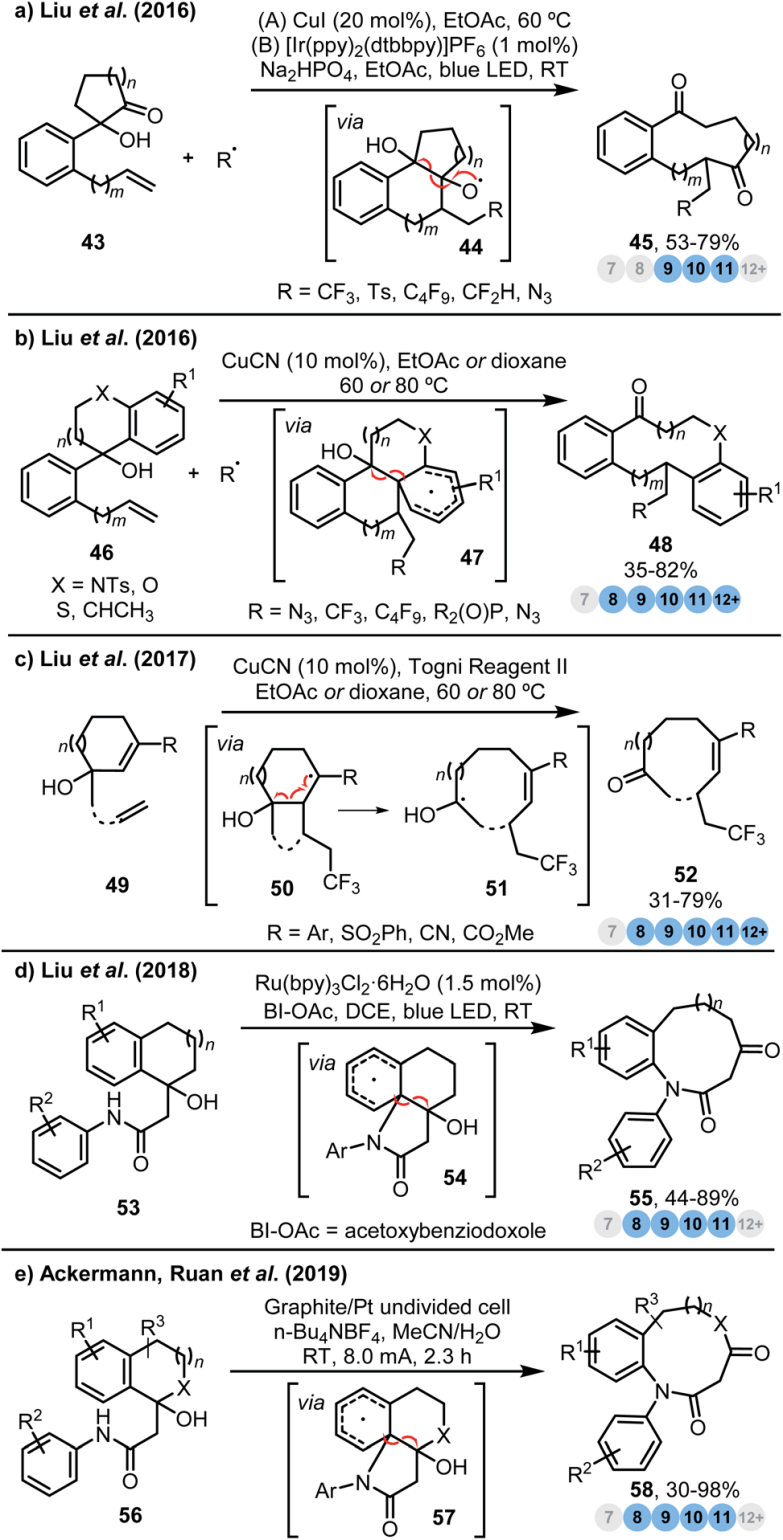
Ring expansion strategies driven by an increase in radical stability. (a) Liu *et al.*^20*b*^ (b) Liu *et al.*^20*b*^ (c) Liu *et al.*^20*c*^ (d) Liu *et al.*^20*d*^ (e) Ackermann, Ruan *et al.*^21^

Radical reactions are also powerful tools for the synthesis of medium-sized rings *via* ring expansion. These reactions are typically driven by an increase in radical stability during the key ring expansion step, with several reaction classes that well illustrate this point having been reported recently by Liu *et al.*^20^ For example, in 2016, this group applied a radical-mediated ring expansion approach in the construction of various benzannulated medium-sized rings (Scheme 5a).^20*a*^ Addition of a range of electrophilic radicals to the alkene moiety of rationally designed alkenyl cyclic α-hydroxyketones **43** triggers ring expansion by a remote 1,4- or 1,5-carbonyl migration process to furnish 9-, 10- and 11-membered rings **45**. The formation of a relatively stable tertiary ketyl radical from a much less stable alkoxy radical **44** is a key driving force for this impressive ring expansion reaction. The same team also reported a novel diversity-oriented synthesis of diverse benzannulated medium-sized rings *via* a conceptually related approach (Scheme 5b).^20*b*^ Again, formation of a relatively stable neutral ketyl radical (from dearomatised radical species **47**) presumably provides a strong driving force for ring expansion. DFT calculations were performed as part of this study, and notable points include: (a) overall the sequential cascade is exothermic by 2.0 kcal mol^−1^ and (b) the presence of an OH group as a π-donating moiety significantly stabilises the resultant ketyl radical species.

In 2017, the Liu group reported a novel C–C bond reorganisation strategy *via* an unprecedented radical 1,3-, 1,4- or 1,5-vinyl migration (Scheme 5c).^20*c*^ Their strategy provides access to a diverse range of fluoroalkyl-containing medium- and large-sized cyclic alkenes **52** (only CF_3_ radical addition products are shown) with excellent chemo-, regio-, and stereoselectivity; the selectivity observed here is particularly impressive given that two alkenyl groups are present in the starting cyclic alkenol substrates **49**. The authors propose that the reaction proceeds *via* selective radical attack at the least sterically hindered terminal alkene to provide a transient alkyl radical which then undergoes *exo* cyclisation to afford radical **50**. Subsequent β-scission and vinyl migration/ring expansion delivers the fluoroalkylated medium-/large-sized cyclic alkenes **52** which is again driven by the formation of another lower-energy neutral ketyl radical **51**.

Most recently, the same group also developed an efficient photocatalytic *n* + 3 ring expansion strategy that provides convenient access to 8–11-membered lactams from readily available cyclic aryl ketones **53**. This variant operates *via* initial generation of a nitrogen centred radical, using visible-light photoredox catalysis (Scheme 5d).^20*d*^ The procedure, which employs a ruthenium-based redox catalyst and acetoxybenziodoxole as an oxidant, exhibits broad scope and the authors were also able to prepare 13–15-membered macrolactams using an *n* → *n* + 4 variant. Although not explicitly mentioned, it is again likely that the driving force of this reaction is the formation of a neutral ketyl radical species **54**.

Finally, Ruan, Ackermann and co-workers reported a catalyst-free, electrochemical variant of this process, which also proceeds *via* a neutral ketyl radical species **57**, including heteroaryl substrates (not shown) and heteroatom tethered species (Scheme 5e).^21^

## Conclusions

The development of any chemical process involving internal rearrangement reactions can be challenging – especially so when the steps have the potential to be reversible. Arguably, these challenges are particularly acute for ring expansion reactions involving the conversion of normal- to medium-sized rings, in view of the thermodynamic penalties often associated with this change in ring size. In this minireview, we have tried to highlight a representative selection of the best and most creative modern methods for the synthesis of medium-sized rings *via* ring expansion methods. In particular, we were keen to place special focus on how the key design aspects of the reactions featured enable the kinetic and thermodynamic challenges associated with medium-sized ring formation to be overcome. Interest in medium-sized rings (and larger ring systems more broadly) in applied fields has undoubtedly increased dramatically in recent years, especially in medicinal chemistry.^4^ As demand for functionalised medium-sized rings grows, so does the need to develop new ways to prepare them, using predictable, practical and scalable methods. We propose that well-designed ring expansion reactions of the type featured in this minireview, are ideally placed to answer this call.

## Conflicts of interest

There are no conflicts to declare.
